# Efficient Sequestration of Hexavalent Chromium by Graphene-Based Nanoscale Zero-Valent Iron Composite Coupled with Ultrasonic Pretreatment

**DOI:** 10.3390/ijerph18115921

**Published:** 2021-05-31

**Authors:** Haiyan Song, Wei Liu, Fansheng Meng, Qi Yang, Niandong Guo

**Affiliations:** 1 Beijing Key Laboratory of Water Resources & Environmental Engineering, China University of Geosciences (Beijing), Beijing 100083, China; song.hy@cugb.edu.cn (H.S.); hnliuzuowei@163.com (W.L.); 18810621792@126.com (N.G.); 2 Chinese Research Academy of Environmental Sciences, Beijing 100012, China; mengfs@craes.org.cn

**Keywords:** graphene-based nanoscale zero-valent iron, ultrasonic pretreatment, hexavalent chromium

## Abstract

Nanoscale zero-valent iron (nZVI) has attracted considerable attention for its potential to sequestrate and immobilize heavy metals such as Cr(VI) from an aqueous solution. However, nZVI can be easily oxidized and agglomerate, which strongly affects the removal efficiency. In this study, graphene-based nZVI (nZVI/rGO) composites coupled with ultrasonic (US) pretreatment were studied to solve the above problems and conduct the experiments of Cr(VI) removal from an aqueous solution. SEM-EDS, BET, XRD, and XPS were performed to analyze the morphology and structures of the composites. The findings showed that the removal efficiency of Cr(VI) in 30 min was increased from 45.84% on nZVI to 78.01% on nZVI/rGO and the removal process performed coupled with ultrasonic pretreatment could greatly shorten the reaction time to 15 min. Influencing factors such as the initial pH, temperature, initial Cr(VI) concentration, and co-existing anions were studied. The results showed that the initial pH was a principal factor. The presence of HPO_4_^2−^, NO_3_^−^, and Cl^−^ had a strong inhibitory effect on this process, while the presence of SO_4_^2−^ promoted the reactivity of nZVI/rGO. Combined with the above results, the process of Cr(VI) removal in US-nZVI/rGO system consisted of two phases: (1) The initial stage is dominated by solution reaction. Cr(VI) was reduced in the solution by Fe^2+^ caused by ultrasonic cavitation. (2) In the following processes, adsorption, reduction, and coprecipitation coexisted. The addition of rGO enhanced electron transportability weakened the influence of passivation layers and improved the dispersion of nZVI particles. Ultrasonic cavitation caused pores and corrosion at the passivation layers and fresh Fe^0^ core was exposed, which improved the reactivity of the composites.

## 1. Introduction

With the rapid development of industrialization, urbanization, and agriculture activities, chromium (Cr) has played a great role both in industrial and agricultural production [1,2]. In industrial production, it is extensively used in electroplating, metal finishing, tannery operations, chemical and battery manufacturing, etc. [3,4]. Waste residue and wastewater with Cr are discharged irrationally, which may be responsible for severe water and soil pollution [5,6]. In agricultural production, pesticides containing Cr are widely used [7,8]. Thus, wastewater with Cr runs into rivers or lakes via surface runoff or into ground water by permeation. The toxicity of chromium is mainly due to the damage of hexavalent chromium (Cr(VI)) in animals and plants [9,10]. It is recognized that Cr(VI) is much more toxic and mutagenic than Cr(III), even at a lower concentration [6,11]. The maximum acceptable levels of Cr(VI) specified by the World Health Organization (WHO) and by the European Union, United States, and Chinese drinking water standards are 50 μg L^−1^, 50 μg L^−1^, 100 μg L^−1^, and 50 μg L^−1^, respectively [12]. Therefore, the uppermost way to remove Cr(VI) from aqueous is to convert Cr(VI) into Cr(III).

In the previous research, there have been several kinds of methods for Cr(VI) removal and remediation, such as chemical, physical, and biological methods [13]. Bioremediation technology has been proven to have good economic benefits and high efficiency. However, functional bacteria reproduce slowly, which makes the remediation term quite long. Adsorption has been considered as an economical and efficient method for Cr(VI) removal. Nevertheless, most absorbents does not have reduction ability [14]. Of all the known methods, sequestration by nanoscale zero-valent iron (nZVI) has been considered as an economic, efficient, and easily implemented process [15], owing to its large specific surface area, high surface reactivity, and low cost [16,17,18]. Cr(VI) can be reduced to Cr(III) by nZVI. Afterward, Cr(III) will form a complex precipitate with OH^−^, Fe^2+^, and Fe^3+^, which can be adsorbed by nZVI and coprecipitated onto nZVI particles.

However, nZVI corrodes easily during the application and storage process [19,20]. Researches have confirmed the “core-shell” structure of iron nanoparticles by spherical aberration-corrected transmission electron microscopy (Cs-STEM) [21]. The iron oxide shell hinders the transfer of electrons, which results in a loss of the activity of nZVI. Moreover, due to its small size, high surface energy, and inherent magnetic character, nZVI is extremely easy to agglomerate, which downsizes the specific surface area and inhibits the interaction of nZVI particles with contaminants [22,23]. Therefore, researchers have attempted to explore methods to avoid these limitations, such as activating nZVI particles before use, modifying with metal, or employing suitable supports [24,25,26].

Graphene, owing to its high electron mobility and large surface area (2630 m^2^ g^−1^) [27,28,29], has attracted wide concern as an absorbent support material. However, the mass production of pristine graphene is hardly achieved because of the difficult bottom-up synthesis. Graphene oxide (GO), which can be synthesized from graphite obtained directly from nature and has a similar hexagonal carbon structure to graphene, has come into public sight [30]. Consequently, as an alternative, reduced graphene oxide (rGO), which could to a certain extent resemble pristine graphene’s properties [31,32] and be easily obtained by reduction of GO [33], has become a preferred support material for absorbent research.

ZVI and nZVI usually need to be activated before use. However, iron oxide can also provide many adsorption sites and has excellent adsorption capacity for pollutants. It is necessary to develop an activation method without removing the iron oxide shell [25]. Ultrasonic technology has gradually developed as a novel and clean pollutant purification method since the 1980s. It has broad prospects due to its low cost, simple control, and potential application in industrial production. Studies have shown that ultrasonic cavitation has the ability of cleaning and activating the passivation layer on the surface of nZVI particles. In a heterogeneous ultrasonic reaction system, microjets rushing to the surface of the solid catalyst caused by cavitation can lead to pores and corrosion on the surface of nZVI particles [34,35].

So as to alleviate the passivation and agglomeration of nZVI and improve the efficiency of Cr(VI) sequestration on nZVI, ultrasonic pretreatment coupled with a graphene-based nanoscale zero-valent iron (US-nZVI/rGO) system was established in this study. GO was used to synthesis the graphene-based nZVI composite because GO can be reduced at the same time as the Fe(III) reduction during the process of nZVI preparation. Ultrasonic pretreatment was used before the reaction to lead to corrosion on nZVI and speed up the reaction of Cr(VI) reduction. Characterizations such as SEM-EDS, BET, XRD, and XPS were performed to investigate the difference in structure between nZVI and nZVI/rGO, and to uncover the mechanism of Cr(VI) removal in the US-nZVI/rGO system.

## 2. Materials and Methods

### 2.1. Preparation of Composites

Graphene oxide used in this study was synthesized using the modified Hummers method [36,37], which is the same method used in the previous study by our group [38]. First, nZVI was synthesized with a classic method of chemical reduction (shown in the Supplementary Materials), as previously reported [39]. When preparing the nZVI/rGO, 0.088 g GO was added into 50 mL deoxy-deionized water and sonicated for 30 min. The amount of GO accounted for 15% of the total of the synthesized composites. Then, the prepared FeCl_3_·6H_2_O aqueous solution was added into the GO suspension and stirred for 30 min with mechanical agitation. The follow-up procedures were similar to those for synthesizing nZVI.

### 2.2. Characterization

Scanning electron microscopy (SEM, Zeiss Merlin) was selected to characterize the surface morphologies, surface composition, and dispersibility of the nanoparticles. Followed that, energy-dispersive X-ray spectroscopy (EDS) was used to determine the element composition. Brunauer, Emmett, and Teller (BET) specific surface area was determined on an automatic specific surface and porosity analyzer (Quantachrome IQ2, Boynton Beach, FL, USA). X-ray diffraction (XRD) spectra were measured using a Bruker AXS D8 Advanced diffractometer with Cu/Kα radiation at 40 kV and 40 mA. The surface compositions of samples before and after reaction were analyzed via X-ray spectroscopy (XPS) using an Al Kα source with a power of 150 W (ESCALAB 250Xi, Thermo Fisher, Waltham, MA, USA).

### 2.3. Bath Experiments

The bath experiments of the Cr(VI) sequestration were conducted in serum bottles (250 mL). A stock solution of Cr(VI) (1000 mg L^−1^) was previously prepared by dissolving potassium dichromate in deionized water. First, 0.2 g nZVI/rGO was added into 200 mL of deoxygenated deionized water. To ensure the anaerobic conditions, deionized water was purged with N_2_ for 10 min before adding nZVI/rGO particles. Second, a required volume of the Cr(VI) stock solution was rapidly injected into the solution and shaken in a thermostatic water bath shaker (SH-A, Changzhou, China) at 180 rpm. For ultrasonic pretreatment experiments, the serum bottles were sealed with rubber plugs and then sonicated for several minutes before adding the Cr(VI) stock solution. The samples were collected and immediately filtered through a 0.22 μm filter with a disposable syringe. The impact of different materials for Cr(VI) removal was investigated by 3 kinds of materials (GO, nZVI, and nZVI/rGO) with the same dosage (0.1 g L^−1^). The ultrasonic pretreatment experiments were studied with 40 kHz ultrasonic frequency. The controlled experiments were carried out under 4 control conditions: Initial pH, system temperature, initial Cr(VI) concentration, and coexisting anions, respectively (Table 1). Moreover, the pH values were adjusted with 0.1 M of HCl and 0.1 M of NaOH. All the experiments were performed in duplicate.

The concentration of Cr(VI) was determined using a ultraviolet-visible (UV) spectrophotometer (752, Shanghai Precision Science Instrument Co., Ltd.) at a wavelength of 540 nm. The concentration of Fe(II) was determined by the phenanthroline spectrophotometric method at a wavelength of 510 nm. The pH value was measured by pH meter (Thermo Scientific, Waltham, MA, USA). The removal capacity of Cr(VI) by nZVI/rGO was calculated with Equation (1). The concentration of Total chromium (*T_Cr_*) and Total iron (*T_Fe_*) were monitored by inductively coupled plasma mass spectrometry (ICP-MS, Agilent 7800, Santa Clara, CA, USA).

(1) $$q_{t}=(\frac{C_{0}{-C}_{t}}{m})V$$

where *C*_0_ (mg/L) is the initial concentration of Cr(VI), *C_t_* (mg/L) is the concentration of Cr(VI) at time *t*, *m* (g) is the amount of nZVI/rGO added into the solution, and *V* (L) is the volume of the solution.

### 2.4. Adsorption Kinetics

The process of Cr(VI) removal on nZVI/rGO from an aqueous solution was described by the pseudo-first-order kinetic model (PFO) [40], pseudo-second-order kinetic model (PSO) [41] and intraparticle diffusion model [42]. The equations of these models are expressed as Equations (2)–(4), as follows:

Linearized PFO kinetic model:

(2) $${\ln(q}_{e}-q_{t}{)=\ln q}_{e}{-k}_{1}t$$

Linearized PSO kinetic model:

(3) $$\frac{t}{q_{t}}=\frac{1}{k_{ad}q_{e}{}^{2}}+\frac{t}{q_{e}}$$

where *q_e_* is the equilibrium adsorption capacity of nZVI/rGO (mg g^−1^), *q_t_* is the adsorption amount at time *t* (mg g^−1^), *t* is the contact time (min), *k*_1_ is the reaction rate constant of the PFO reaction (min^−1^), and *k_ad_* is the reaction rate constant of the PSO reaction (g mg^−1^ min^−1^).

Intraparticle diffusion model:

(4) $$q_{t}=k_{dif}t^{0.5}+C$$

where *k_dif_* is the intraparticle diffusion constant (mg g^−1^ min^−0.5^) and *C* is related to the boundary thickness effect (mg g^−1^).

## 3. Results and Discussion

### 3.1. Cr(VI) Removal by GO, nZVI and nZVI/rGO

**Figure 1:**
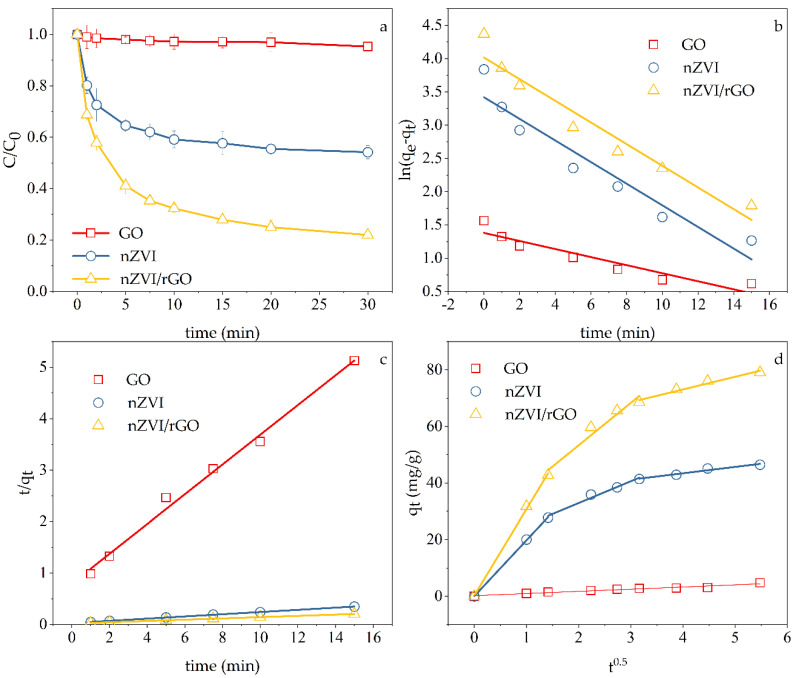
(**a**) Performance on Cr(VI) removal by different materials, (**b**) PFO models, (**c**) PSO models, and (**d**) intraparticle diffusion models. Experimental conditions: Temperature, 30 °C; pH = 5, initial concentration, 10 mg L^−1^; dosage of adsorbents, 0.1 g L^−1^.

Figure 1 and Table 2 and Table 3 depict the effects of different materials on Cr(VI) removal efficiency. The results showed that nZVI presented a much lower removal efficiency than nZVI/rGO. nZVI/rGO obtained the highest removal efficiency of 78%. GO presented the poorest performance on Cr(VI) removal. GO had nearly no adsorption effect on Cr(VI) because of the negative charge brought by the oxygen-containing functional groups on the surface of GO [43]. 

Compared to the pseudo-first-order kinetic (PFO), the process of Cr(VI) removal by nZVI/rGO was better fitted by the pseudo-second-order kinetic (PSO) model with a high coefficient of determination (*R*^2^ = 0.9995), which indicated that the rate-limiting step of this process was chemical adsorption rather than physical diffusion and there might be electrons sharing or transfer between Cr(VI) and nZVI/rGO [44]. Moreover, the theoretical equilibrium adsorption capacity (*q_e_*) calculated based on PSO model was more consistent with the actual experimental situation.

Each of the multilinear curves simulated by the intraparticle diffusion model included three portions. This showed that the adsorption of Cr(VI) on nZVI/rGO was a process with three different stages, which was similar to the process on nZVI, including the transport of Cr(VI) from the aqueous solution to the surface of absorbents (nZVI/rGO), the adsorption onto the absorbents, and the equilibrium adsorption [44,45]. In addition, the curves that did not pass through the origin indicated that the intraparticle diffusion was not the only adsorption mechanism of Cr(VI) anions onto nZVI and nZVI/rGO.

In accordance with the half-reactions (Equations (5) and (6)), it takes 3 mol electrons to reduce 1 mol Cr(VI) to Cr(III), while 1 mol Fe(0) can offer 2 mol electrons by being oxidized to Fe(II). In theory, it was sufficient for the electrons provided by 0.1 g/L nZVI to reduce 10 mg/L Cr(VI) (200 mL solution). However, in Figure 1, nZVI only presented 45.84% efficiency for Cr(VI) removal. The phenomenon might exist because as the reaction proceeded, the passivation layer composed of (hydro)oxides of Cr(III) and Fe(III) continued to generate, which interfered with the electron transfer between nZVI and Cr(VI) and therefore inhibited the subsequent reaction [46]. By comparison, only 85% of the total mass was accounted for nZVI in the nZVI/rGO composites even though the nZVI/rGO presented nearly 80% efficiency for Cr(VI) removal, which demonstrated the specific role of graphene in this process. Graphene has a particularly strong capability of electron transfer. Through graphene, the electrons in the Fe^0^ core can penetrate the passivation layer and cause electron-accepting reactions by Cr(VI) on the graphene.

(5) $${\mathrm{Cr}}^{6+}{+3e}^{-}\to {\mathrm{Cr}}^{3+},$$

(6) $${\mathrm{Fe}}^{0}-{2e}^{-}\to {\mathrm{Fe}}^{2+}$$

### 3.2. Ultrasonic Pretreatment

**Figure 2:**
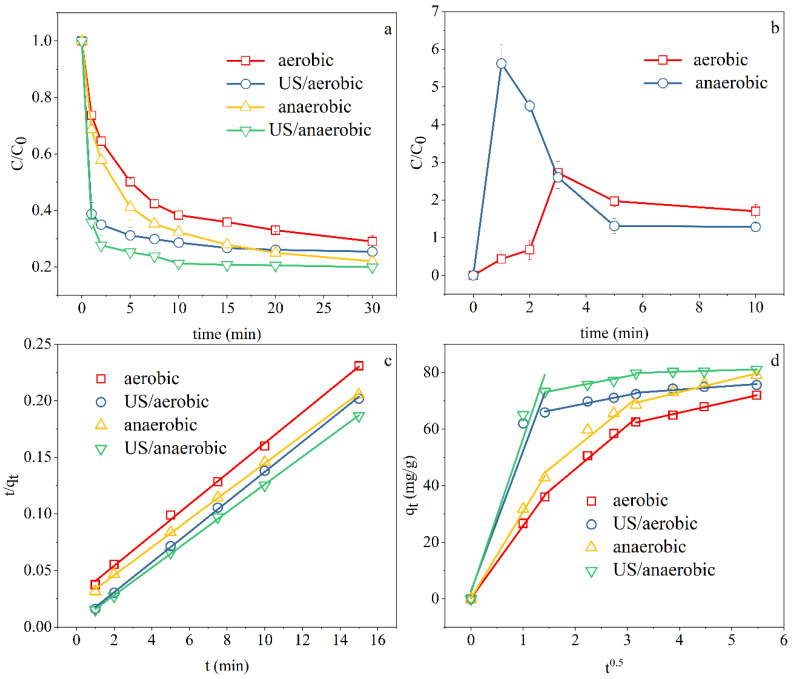
(**a**) Effects of ultrasonic pre-reatment: Removal efficiency, (**b**) concentration of Fe(II) in solution during sonication, (**c**) PSO model fitting results, and (**d**) intraparticle diffusion model fitting results. Experimental conditions: Temperature, 30 °C; pH = 5; initial concentration, 10 mg L^−1^; dosage of nZVI/rGO, 0.1 g L^−1^; ultrasonic frequency = 40 kHz.

The ultrasonic method has wide prospects due to its low cost, simple control, and potential application in industrial production. So as to study the effect of ultrasonic (US) pretreatment coupled with nZVI/rGO for Cr(VI) sequestration, we conducted control experiments with and without ultrasonic pretreatment. As shown in Figure 2a, sonication could effectively speed up the reaction rate. When pretreated with ultrasound, the process of Cr(VI) removal achieved equilibrium within 15 min. In the early stages of the process, the reaction rate was quite rapid, with approximately 65% removal efficiency within 1 min, whereas the two systems without ultrasonic pretreatment had not reached equilibrium by 30 min. In addition, the efficiency of Cr(VI) removal by nZVI/rGO increased slightly from 72.94% and 78.15% to 74.57% and 80.05% for aerobic and anaerobic conditions, respectively.

According to the kinetic fitting results (Figure 2c and Table S1), the processes with ultrasonic pretreatment were still well fitted by the PSO model after 1 min. The curves simulated by the intraparticle diffusion model also included three portions (Figure 2d) which were similar to that obtained without ultrasonic pretreatment. However, the fitting results (Table 4) of the first stage (0~2 min) of the US-nZVI/rGO system was not ideal. The coefficients (*R*^2^) were 0.8903 (aerobic) and 0.9311 (anaerobic), respectively, which were lower than the *R*^2^ of the system without US (0.9987 (aerobic) and 0.9986 (anaerobic)). The point at 1 min was above the simulating curves, which proved that the inflection point between the first stage and the second stage occurred within 2 min. Nevertheless, we regret that we could hardly acquire more compact experimental data because of the short reaction time. Moreover, the *k_dif_* of the first stage in the US-nZVI/rGO system increased compared with that in the system without ultrasonic pretreatment, indicating that the adsorption rate of the first stage increased in the US-nZVI/rGO system. Ultrasonic cavitation makes the membrane diffusion between nZVI/rGO and the aqueous solution much easier.

By monitoring the concentration of Fe(II) in the solution (Figure 2b), a large amount of Fe^2+^ ions was found in the anaerobic system after 1 min of sonication. The Fe(II) in the system played a pivotal role in the sequestration of Cr(VI) as it not only participated in the reduction process of Cr(VI), but also made the surface of nanomaterials carry more positive charge, thereby accelerating the adsorption of negatively charged chromate [47]. Therefore, it can be inferred that there might be a solution reaction process at the very beginning of the ultrasonic pretreatment-coupled system to remove Cr(VI). During the whole reaction phase, both the solution reaction and the interfacial reaction existed simultaneously. Ascribable to the effect of ultrasonic cavitation, small gaps or pores emerged at the passivation layer of nZVI, which exposed the Fe^0^ core to the solution, thus generating a large amount of Fe^2+^ and speeding up the removal of Cr(VI).

### 3.3. Reactivity Test of the Ultrasonic Pretreatment Coupled nZVI/rGO System for Cr(VI) Removal

#### 3.3.1. Effect of Initial pH

**Figure 3:**
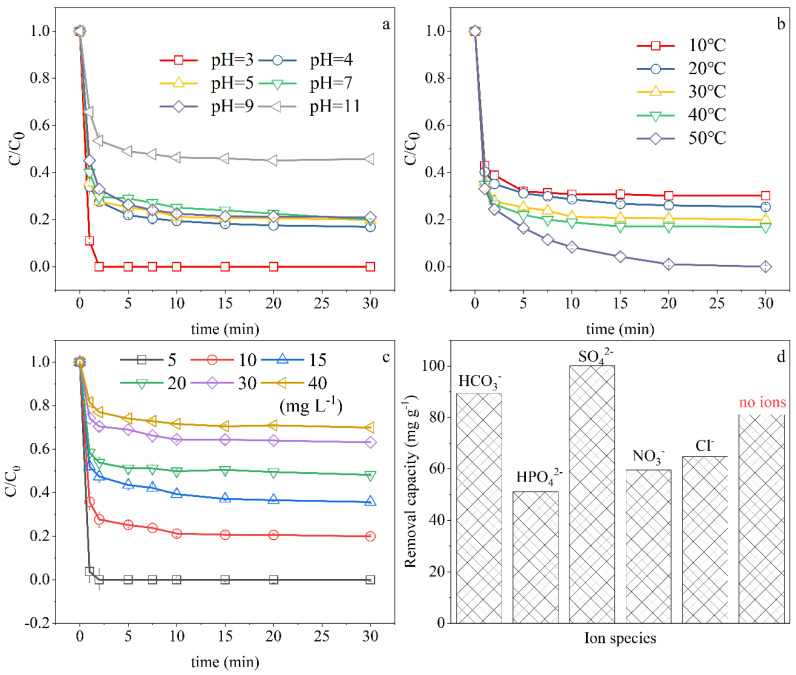
Effects of environmental factors: (**a**) Initial pH, (**b**) temperature, (**c**) initial Cr(VI) concentration, and (**d**) coexisting anions. Fixed experimental conditions: Temperature, 30 °C; pH = 5; initial concentration, 10 mg L^−1^; dosage of nZVI/rGO, 0.1 g L^−1^; ultrasonic frequency = 40 kHz.

pH is one of the most important characteristics of wastewater. It plays an important role in most technologies of wastewater treatment. Therefore, it is necessary to study the influence of the initial pH on Cr(VI) removal by nZVI/rGO in an aqueous solution. Figure 3a depicts the effect of the initial solution pH on the sequestration of Cr(VI). The removal efficiency declined continuously as the initial solution pH increased. The capacity of Cr(VI) removal increased sharply when pH < 4 and decreased sharply when pH > 9, while it varied slightly between pH 4–9. Even at pH 9, the Cr(VI) removal efficiency can reach 81.99%. Compared with nZVI [48], the nZVI/rGO composite increased the tolerance to acid and alkali conditions in the Cr(VI) removal experiments. The existence of Cr(VI) can be affected by the pH value and chromium concentration in the aqueous solutions. When the pH of an aqueous solution remains below 1, Cr(VI) exists as H_2_CrO_4_, and when the pH of an aqueous solution varies between 1 and 6, Cr(VI) exists as the anion of HCrO_4_^−^. When the pH of an aqueous solution is above 6, the form of Cr(VI) changes to the anion of CrO_4_^2−^ [14,49]. As in the previous research [50,51], nZVI particles have a zero-point charge (pHzpc) around a pH of 7~8. Nanoparticles acquire a positive point charge below pHzpc and a negative point charge above pHzpc. When the reaction was conducted at a pH below the pHzpc of nZVI/rGO, the Cr(VI) anions HCrO_4_^−^ with a negative charge could be easily adsorbed onto the surface of nZVI/rGO with a positive charge. The lower the pH, the more Fe(II) cationic will be dissociated from the surface of nZVI/rGO, and the faster the rate of adsorption caused by electrostatic interaction will be.

#### 3.3.2. Effect of Temperature

The effect of temperature on Cr(VI) removal is shown in Figure 3b. When the temperature increased from 10 °C to 50 °C, the removal efficiency at equilibrium grew from 70% to 100%. With the increase of temperature, the movement of each component in the reaction system was accelerated, which accelerated the diffusion rate of Cr(VI) from the solution to the surface of adsorbents and promoted the binding of Cr(VI) to the active sites on the composites [52]. However, as shown in Figure 3b, the influence of the reaction temperature was not appreciable, especially at the very beginning of the process. When the temperature rose from 10 °C to 40 °C, the removal efficiency at 30 min only increased by 10%. When the temperature rose from 40 °C to 50 °C, the variation of removal efficiency was particularly significant with an increase of 20%. The results demonstrate that the application of nZVI/rGO could be carried out in a wide temperature range. 

#### 3.3.3. Effect of the Initial Cr(VI) Concentration

The initial Cr(VI) concentrations with 5 mg L^−1^, 10 mg L^−1^, 15 mg L^−1^, 20 mg L^−1^, 30 mg L^−1^, and 40 mg L^−1^ were investigated in order to understand their effects on the removal performance. Figure 3c showed that the corresponding removal efficiency of Cr(VI) at 30 min was 100%, 80.05%, 58.36%, 49.44%, 36.40%, and 26.21%, respectively. To further investigate the effect of the initial concentration on the Cr(VI) removal, PSO kinetic analysis was performed. As shown in Figure S1 and Table S2, the equilibrium removal capacity kept an upward tendency as the initial concentration of Cr(VI) increased from 10 mg L^−1^ to 20 mg L^−1^. However, it dropped significantly when the initial concentration of Cr(VI) increased to 30 mg L^−1^. Many studies have also reported that the increase of the initial concentration can easily lead to a reduction of the removal efficiency. As known, Cr(VI) is an oxidant, which can effectively passivate nZVI. The main reason for the reduction of the removal efficiency is the passivation effect of Cr(VI) on the nZVI surface. With the increase of the Cr(VI) and nZVI concentration ratio, the amount of Cr(VI) around nZVI increased, leading to the accelerated oxidation of nZVI. Subsequently, a large amount of iron and chromium (hydro)oxides such as (Cr_x_Fe_1−x_)(OH)_3_ and Cr_x_Fe_1−x_OOH were deposited on the surface of nZVI and graphene, which impeded the further reaction of nZVI/rGO with Cr(VI). In addition, the dosage of nZVI was fixed and the available active sites remained unchanged, resulting in a decrease in the percentage of Cr(VI) removal as the initial concentration increases [53].

#### 3.3.4. Effect of Coexisting Anions in the Solution

Natural waters and wastewater are both complex systems. Because of the high reactivity of nZVI/rGO, there are many components which can strongly affected the removal efficiency of Cr(VI) on nZVI/rGO. For this reason, this paper studied the effects of the presence of several common inorganic anions, such as HCO_3_^−^, HPO_4_^2−^, SO_4_^2−^, Cl^−^, and NO_3_^−^, on the reactivity of nZVI/rGO to remove Cr (VI) in water. In order to eliminate the influence of different cations, the inorganic salts used in the experiment were sodium salts of the same cation, and the equivalent ion concentration (1 mmol L^−1^) was selected. As shown in Figure 3d, when these five species of anions separately existed in aqueous solution, the removal efficiency was 88.15%, 50.35%, 98.73%, 58.76%, and 63.86%, respectively. The presence of HPO_4_^2−^, NO_3_^−^, and Cl^−^ had a strong inhibitory effect on this process, among which HPO_4_^2−^ showed the poorest appearance. The equilibrium adsorption capacity (*q_e_*) of nZVI/rGO in these three solutions was 51.03 mg g^−1^, 59.56 mg g^−1^, and 64.73 mg g^−1^, respectively, which decreased by 37.10%, 26.59%, and 20.22% compared to the *q_e_* (81.14 mg g^−1^) of the single nZVI/rGO system. The strong inhibition of phosphates is due to the competition with Cr(VI) in the reaction system. Phosphates can be adsorbed by nZVI/rGO composites and precipitate with Fe^2+^ or Fe^3+^ by forming Fe_3_(PO_4_)_2_ or FePO_4_ [54,55]. The existence of HCO_3_^−^ and SO_4_^2−^ can promote the efficiency of Cr(VI) removal, of which the effect of SO_4_^2−^ was more obvious. The q_e_ (100.06 mg g^−1^) of nZVI/rGO in the SO_4_^2−^-containing system was about 20% higher than that of the single nZVI/rGO system. SO_4_^2−^ is a low-affinity ligand which can form outer-sphere complexes with iron hydroxide or carobnyl [56]. Outer-sphere complexation has a weak effect. Therefore, the competition with Cr(VI) during the reaction can be negligible. The facilitation of SO_4_^2−^ might be attributed to the increase of ionic strength in the solution. The appropriate increase of ionic strength can aggravate the corrosion of Fe^0^ and thus accelerate the reaction between Cr(VI) and Fe^0^ [57]. 

### 3.4. Characterization

#### 3.4.1. SEM-EDS and BET Analysis

**Figure 4:**
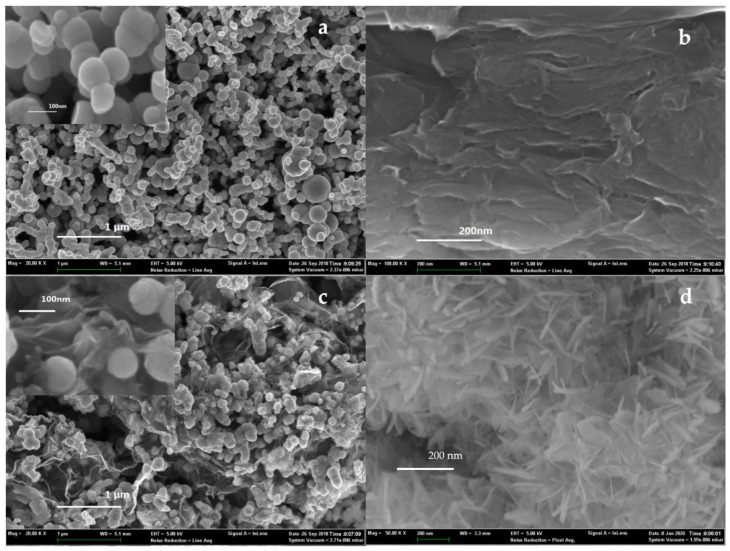
SEM images of (**a**) nZVI, (**b**) GO, (**c**) nZVI/rGO, and (**d**) solids obtained after reaction.

The SEM of nZVI, GO, nZVI/rGO, and the solids obtained after the reaction are shown in Figure 4. The nZVIs synthesized in this study were spherical particles, which had diameters varying from 10–100 nm, and were aggregated into chains under magnetic force and electrostatic force (Figure 4a). The structure of the GO was nanosheets with folds (Figure 4b). After loading onto graphene, the single nZVI particles were dispersed in the folds of graphene (Figure 4c), which improved the dispersion of the nanoparticles. After use, the spherical particles symbolizing nZVI disappeared. Instead, needle-like solids appeared on the surface of composite (Figure 4d), which were presumed to be iron (hydro)oxides.

The EDS spectra can qualitatively and semi-quantitatively analyze the element distribution of the outer layer of materials. As shown in Figure S2 and Table S3, the nZVI had attached to the surface of the rGO, and the mass ratio of nZVI to GO was close to the theoretical value. 

According to the previous references, the BET specific area of the bare nZVI was around 14–35 m^2^/g (Table S5). In accordance with the results of the BET (Figure S3 and Table S4), the specific area of the GO and nZVI/rGO was 1.23 m^2^/g and 59.31 m^2^/g, respectively. Apparently, although the specific area of the new composite made little improvement compared with the bare nZVI, dispersibility had been developed. It has also been demonstrated that nZVI/rGO composite has a typical mesoporous structure [53,54]. Based on the BJH(Barrett-Joyner-Halenda) desorption technology, the average pore size of nZVI/rGO was 2.324 nm.

#### 3.4.2. XRD Analysis

**Figure 5:**
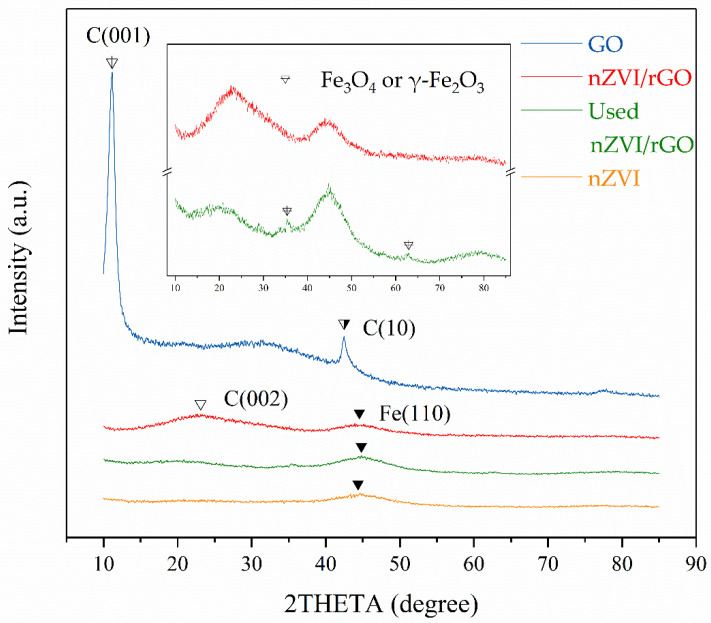
XRD patterns of GO, nZVI, nZVI/rGO, and the used nZVI/rGO.

Figure 5 showed the wide-angle X-ray diffraction (XRD) patterns of the GO, nZVI, nZVI/rGO, and the used nZVI/rGO. GO exhibited a diffraction peak centered at 2θ = 11.16° (001), which indicated that GO had a typical lamellar structure [58]. During the preparation of nZVI/rGO, GO was reduced to rGO by NaBH_4_. As a consequence, the (001) peak of GO disappeared and was alternatively replaced by a diffraction peak at 2θ ≈ 23.12°, which corresponds to the crystal plane of graphite (002) [59]. The ideal (002) crystal plane of graphite presented at 2θ = 26.4°. In this study, the peak shift of the (002) crystal plane occurred because the rGO produced by chemical reduction was not completely reduced and there were still a few oxygen-containing functional groups between the layers or at the edges of the layers. Similar results were observed in the research of Stobinski et al. [60]. They used hydrazine hydrate to reduce GO, and the (002) peak of the obtained rGO occurred at 2θ = 23.76°.

As observed in Figure 5, the nZVI showed an obvious reflection peak at 2θ = 44.36°, belonging to the characteristic peak (110) of the cubic Fe (JCPDS, file no. 06-0696), which was easily oxidized when exposed to air due to its high activity with a large specific surface area. A strong reflection peak also appeared at 2θ = 44.53° on the curve of nZVI/rGO, similar to the peak of nZVI, which means that the nZVI was successfully loaded on the layer of rGO. In addition to the (110) peak detected at 2θ = 44.8°, weak diffraction peaks of 2θ = 35.42° and 2θ = 62.94° were detected in the XRD pattern of the used nZVI/rGO, indicating the presence of Fe_3_O_4_ or γ-Fe_2_O_3_ [61,62].

#### 3.4.3. XPS Analysis

An XPS analysis was performed to provide insight into the actual components on the surface of nZVI/rGO before and after the reaction. Figure S5 exhibited the full XPS spectra of nZVI/rGO and GO after reaction. As shown in Figure S4, photoelectron lines appeared at binding energies of 285.6 eV, 532.6 eV, and 712.6 eV, which belonged to C 1 s, O 1 s, and Fe 2p, respectively. The results prove that Fe(0) was successfully adapted onto the surface of rGO. 

**Figure 6:**
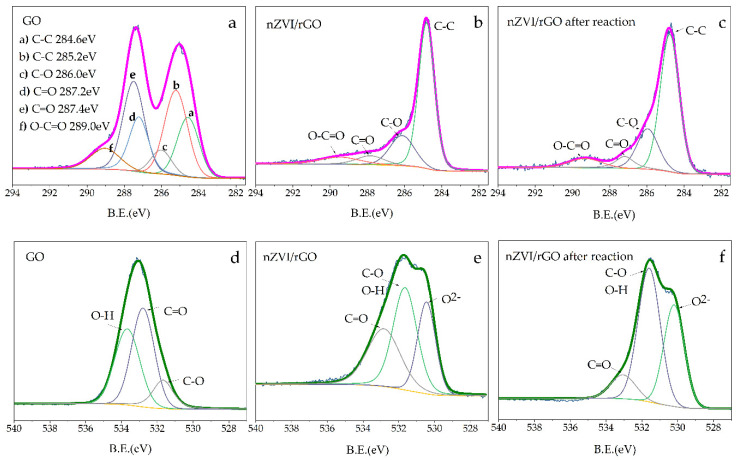
High-resolution XPS spectrum of (**a**–**c**) C 1s and (**d**–**f**) O 1s of GO, nZVI/rGO, and nZVI/rGO after reaction.

Moreover, the narrow region XPS spectra of each single element was studied to develop their transformation during the experiments. The spectra of C 1 s are shown in Figure 6a–c. The spectra apparently indicate that there were different carbon atoms in four different functional groups on the surface of GO, as follows: The nonoxygenated ring carbon (C–C), the carbon in C–O bonds, the carbonyl C (C=O), and the carboxylate carbon (O–C=O), whose photoelectron peaks usually appeared at around 284.6 eV, 286.0 eV, 287.8 eV, and 289.0 eV, respectively [63]. Although there were also peaks of these oxygen functional groups in the C 1s XPS spectrum of nZVI/rGO, the intensity was much weaker than those in GO. Then, we integrated the spectra to obtain the area of each peak. The results (Table S6) showed that the peak area of C-C groups amounted to 44.06%, which increased to 67.45% after reduction. The amount of oxygen-containing groups decreased from 55.94% to 38%, which demonstrates that most of the oxygen functional groups were eliminated during the reduction of Fe(III). Graphene is known to be highly conductive, mainly because of the long-range conjugated network in the graphitic lattice [63,64]. Nevertheless, the existence of functional groups could break the conjugated network and localize the π-electrons. Consequently, the aim of reducing GO is not only to remove the oxygen functional groups bonded to graphene and other atomic-scale lattice defects but also to recover the conjugated network [63]. Then, the electrical conductivity of the graphene would be recovered through the reduction of GO.

Figure 6d–f show the high-resolution XPS spectrum of O 1s of GO and nZVI/rGO. The O 1s spectrum of the GO could be deconvoluted into three peaks, which were attributed to C–O (531.7 eV), C=O (532.8 eV) [50], and O–H groups of adsorbed H_2_O (533.7 eV) [65]. The O 1s spectrum of nZVI/rGO could also be fitted by three types of oxygen species, which were contributed to the oxygen-containing atom groups O–H, C–O (531.7 eV), C=O (532.8 eV), and O^2−^ (530.4 eV) in the oxide layers of iron, respectively [66]. Compared with these two spectra, the amount of C=O declined, and C–O–Fe may have formed on the surface of GO during the process of reduction by NaBH_4_.

### 3.5. Mechanism Analysis

In order to study the mechanism of Cr(VI) removal from an aqueous solution by nZVI/rGO, the XPS spectrum was further developed. As can be seen in Figure S4, the photoelectron peaks of Cr 2p were detected in the full survey pattern of nZVI/rGO after the reaction, which illustrates that chromium was adsorbed onto nZVI/rGO. The C 1s spectrum of nZVI/rGO after the reaction (Figure 6c) was similar to that of nZVI/rGO, which means that the reduction of Cr(VI) had little effect on the surface functional groups of nZVI/rGO. As described in Figure 6f, the amount of OH^−^ (531.6 eV) increased significantly after the reaction, which provided the conditions to form chromium oxide or hydroxide.

**Figure 7:**
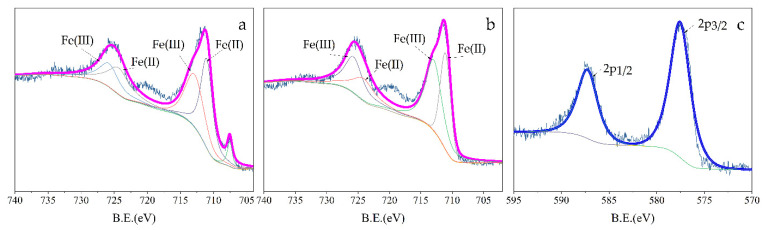
High-resolution XPS spectrum of Fe2p and Cr2p: (**a**) nZVI/rGO, (**b**) nZVI/rGO after reaction, and (**c**) Cr2p after reaction.

Figure 7a,b display the high-resolution XPS spectrum of Fe 2p. Compared with the two spectra, Fe(0) (707.5 eV) was detected as a weak signal in unused nZVI/rGO, which indicates that there might have been an oxide layer wrapping on the surface of the nZVI because the photoelectrons could only be detected by XPS from the outer surface of 10 nm [50]. Moreover, the photoelectron peaks at binding energies 710.9 eV and 724.2~724.5 eV were attributed to Fe(II), while the peaks at 712.7~712.8 eV and 725.9 eV were ascribed to Fe(III) [67]. A large amount of Fe(II) still existed on the surface because of the enrichment of Fe(II) by nZVI/rGO [68]. In conclusion, there might have been two processes that happened on Fe species during the Cr(VI) reduction, namely: (a) Fe(0) could have reacted with Cr(VI) directly, and (b) Fe(II) could have reduced Cr(VI) into Cr(III). Furthermore, the valence states of the Cr species on nZVI/rGO after the reaction were analyzed by the narrow region XPS spectrum of Cr 2p in Figure 7c. The two peaks corresponding to the Cr 2p1/2 and Cr 2p3/2 could be deconvoluted into two doublets. The peaks at binding energies of 577.7 eV and 587.1 eV, between which there was a firm distance of 9.4 eV, were attributed to Cr(III) [69,70,71]. There were no obvious photoelectron peaks of Cr(VI) from which we could deduce that most Cr(VI) had been reduced to Cr(III).

It is well known that nZVI is inevitably oxidized during the preparation and repositing process, which can result in the formation of a passivation layer and restrain the contact of contaminant and nZVI. During this research, we found that the passivation of nZVI in the nZVI/rGO composite was distinctly alleviated. In order to prove this, we used the samples of nZVI/rGO, which had been kept for 1, 6, and 12 months after preparation to conduct the experiment of Cr(VI) sequestration. The results showed that even the oldest sample had a removal efficiency of around 70% (Figure S5). This might be because graphene has good electron transfer ability, which could help the electrons from nZVI ”penetrate” the passivation layer and arrive at Cr(VI) anions. Furthermore, the electrons entering the reaction system through graphene could also reduce the Fe(III) in the passivation layer to Fe(II) by a neutralization reaction, which could eliminate the passivation layer and provide a new electron transportation aisle for the nZVI. The obtained Fe(II) could be used to continue reducing Cr(VI), and thus facilitate in the process of the reduction reaction. Under acid conditions, nZVI and rGO could form a primary battery, in which nZVI performs as the anode and rGO as the cathode [50]. The electrons were transferred over the surface of the nZVI/rGO materials and between nZVI and rGO.

**Figure 8:**
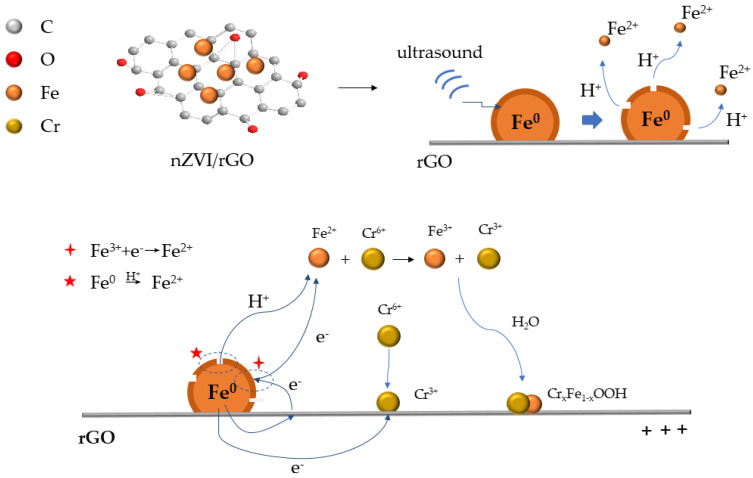
Mechanism of the sequestration of Cr(VI) on nZVI/rGO coupled with ultrasonic pretreatment.

Based on the above results, the chemical reactions occurring in the system can be summarized as follows (Figure 8):

(7) $${\mathrm{Cr}}_{2}O_{7}{}^{2-}{+14H}^{+}+6e\;\to {\;2\mathrm{Cr}}^{3+}{+7H}_{2}O\;E_{\mathrm{Cr}(\mathrm{VI}),\mathrm{Cr}(\mathrm{III})}=1.33V$$

(8) $$3\mathrm{Fe}\;-\;6e\;\to {\;3\mathrm{Fe}}^{2+}\;E_{{\mathrm{Fe}}^{2+},\mathrm{Fe}\;}=-1.32V$$

(9) $${2\mathrm{Fe}}^{3+}{+\mathrm{Fe}}^{0}\;\to {\;3\mathrm{Fe}}^{2+}\Delta E=2.21\mathrm{Ev}$$

(10) $${\;\mathrm{Cr}}_{2}O_{7}{}^{2-}{+6\mathrm{Fe}}^{2+}{+14H}^{+}\;\to {\;6\mathrm{Fe}}^{3+}{+2\mathrm{Cr}}^{3+}{+7H}_{2}O$$

(11) $${\mathrm{xCr}}^{3+}{+(1-x)\mathrm{Fe}}^{3+}{+2H}_{2}O\;\to {\;\mathrm{Cr}}_{x}{\mathrm{Fe}}_{\left(1-x\right)}{\mathrm{OOH}(s)+3H}^{+}$$

When ultrasound was applied, a high-energy source was generated, which caused cavitation. Acoustic cavitation is a process in which bubble generation, maturation, and implosion occur simultaneously in a liquid environment. The last moment of bubble burst can form a liquid jet with a high speed of about 111 m/s, which is also known as a shockwave [72]. Under this impact, the passivation layer could partly fall off and form a gap on the surface of nZVI. Meanwhile, part of the Fe(0) core could be freshly exposed and improve the reactivity of nZVI (Figure 8). Additionally, because of the large specific surface area of graphene, quantities of cavitation bubbles were adsorbed onto the composites, which strengthened the process. Moreover, at the instant of cavitation bubble burst, extremely high temperature and pressure were generated, as well as rapid heating and cooling rates [73], which could be a positive drive of chemical reactions.

## 4. Conclusions

An efficient nZVI/rGO composite for the removal of Cr(VI) from an aqueous solution was produced in this research. Compared with nZVI, the introduction of rGO could help alleviate the passivation and agglomeration of nZVI. Ultrasonic pretreatment on the nZVI/rGO composite was implemented before the reaction. The addition of ultrasonic pretreatment efficiently shorted the adsorption time and speeded up the remove rate of Cr(VI) removal, as proven by the simulating result of the intraparticle diffusion model. Moreover, acid pH value and high temperature played a catalytic role in Cr(VI) removal. The presence of SO_4_^2−^ and HCO_3_^−^ accelerated the sequestration of Cr(VI) in US-nZVI/rGO system, while the presence of HPO_4_^2−^, NO_3_^−^ and Cl^−^ had an inhibitory effect on this process. The mechanism of Cr(VI) removal on nZVI/rGO coupled with ultrasonic pretreatment can be concluded as: Cr(VI) first reacted with Fe(II) generated by the ultrasonic cavitation, Cr(VI) was reduced on the surface of nZVI/rGO, and Cr(III) (hydro)oxides were adsorbed and coprecipitated on rGO. nZVI/rGO coupled with ultrasonic pretreatment is a promising method for the removal of Cr(VI) and many other kinds of heavy metals and organics from an aqueous solution. The US-nZVI/rGO system can also be applied in some special situations which need to be solved in a very short time, such as an emergency in a factory.

## Figures and Tables

**Table 1:** Controlled conditions of the batch experiments (dosage of adsorbents 0.1 g L^−1^; ultrasonic frequency 40 kHz).

Influence Factors	Conditions
Initial pH	3, 4, 5, 7, 9, 11
System temperature	10 °C, 20 °C, 30 °C, 40 °C, 50 °C
Initial Cr(VI) concentration	5, 10, 15, 20, 30 mg·L^−1^
Coexisting anions	Cl^−^, SO_4_^2−^, NO_3_^−^, HCO_3_^−^, HPO_4_^2−^

**Table 2:** PFO and PSO kinetic parameters for different materials.

Kinetic Model	Material	Parameter		
PFO		*k*_1_ (min^−1^)	*q_e_* (mg g^−1^)	*R* ^2^
	GO	0.061	3.98	0.8865
	nZVI	0.16	30.56	0.9221
	nZVI/rGO	0.16	55.53	0.9441
PSO		*k_ad_* (g mg^−1^ min^−1^)	*q_e_* (mg g^−1^)	*R* ^2^
	GO	0.10	3.46	0.9928
	nZVI	0.015	46.84	0.9994
	nZVI/rGO	0.0072	80.91	0.9996

**Table 3:** Parameters of the intraparticle diffusion models for different materials.

Material	The First Stage		The Second Stage		The Third Stage	
	*k_dif_* (mg g^−1^ min^−0.5^)	*R* ^2^	*k_dif_* (mg g^−1^ min^−0.5^)	*R* ^2^	*k_dif_* (mg g^−1^ min^−0.5^)	*R* ^2^
nZVI	19.73	0.9998	7.70	0.9805	2.26	0.9618
nZVI/rGO	30.51	0.9986	15.01	0.9567	4.48	0.9673
GO	*k_dif_* = 0.7665			*R*^2^ = 0.9550		

**Table 4:** Parameters of the intraparticle diffusion models in different systems.

System	The First Stage		The Second Stage		The Third Stage	
	*k_di_*_f_ (mg g^−1^ min^−0.5^)	*R* ^2^	*k_dif_* (mg g^−1^ min^−0.5^)	*R* ^2^	*k_dif_* (mg g^−1^ min^−0.5^)	*R* ^2^
Aerobic	25.63	0.9973	15.48	0.9882	4.15	0.9969
US/Aerobic	49.44	0.8903	3.68	0.9799	1.33	0.8749
Anaerobic	30.51	0.9972	15.01	0.9567	4.48	0.9673
US/Anaerobic	54.27	0.9311	3.58	0.9685	0.54	0.9781

## Supplementary Materials

The following are available online at https://www.mdpi.com/article/10.3390/ijerph18115921/s1, Figure S1: The fitted curves of the PSO model for US-nZVI/rGO system, Figure S2: EDS spectra of (a) nZVI/rGO and (b) nZVI/rGO after reaction, Figure S3: Nitrogen adsorption-desorption isotherms and pore size distribution curve (the insert pattern) for nZVI/rGO, Figure S4: Full survey XPS patterns for nZVI/rGO, nZVI/rGO after reaction, and GO, Figure S5: Influence of storage time, Table S1: PSO kinetic parameters of nZVI/rGO coupled with ultrasonic pre-treatment, Table S2: Parameters obtained from PSO kinetic model for US-nZVI/rGO system, Table S3: The mass ratio of each element from EDS, Table S4: Physical and chemical properties of nanoparticles investigated in this study, Table S5: BET specific surface area of nZVI in the provious studies, Table S6: The peak area fraction of carbon-containing groups.
