# Supplementary material for: Efficient Sequestration of Hexavalent Chromium by Graphene-Based Nanoscale Zero-Valent Iron Composite Coupled with Ultrasonic Pretreatment
Source: Int J Environ Res Public Health. 2021 May 31;18(11):5921. doi: 10.3390/ijerph18115921 (PMC8197979; doi:10.3390/ijerph18115921)
Supplement: Supplementary file 1 [file ijerph-18-05921-s001.zip › ijerph-1196138-supplementary.pdf]

Supplementary materials

Efficient sequestration of hexavalent chromium on graphene-based nanoscale zerovalent iron composite coupling with ultrasonic pre-treatment

Haiyan Song [1], Wei Liu [1], Fansheng Meng [2], Qi Yang [1,*] and Niandong Guo [1]

[1] School of Water Resources and Environment, Beijing Key Laboratory of Water Resources and Environmental Engineering, and Environmental Engineering, and MOE Key Laboratory of Groundwater Circulation and Environmental Evolution, China University of Geosciences (Beijing), Beijing 100083, PR China

[2] Chinese Research Academy of Environmental Sciences, Beijing 100012, PR China

* Correspondence: yq@cugb.edu.cn (Q. Y.)

1. Materials and methods

1.1 Materials

All of the chemicals used in the study were of analytical grade. Iron(III) chloride hexahydrate ($FeCl_3 \cdot 6H_2O$), sodium borohydride ($NaBH_4$), potassium dichromate ($K_2Cr_2O_7$), acetone (CH_3COCH_3), and diphenylcarbazide ($C_6H_5(NH)_2CO(NH)_2C_6H_5$) were purchased from Sinopharm Chemical Reagent Co. Ltd., China. Hydrochloric acid (HCl, 36%-38%), sodium hydroxide (NaOH), phosphoric acid (H_3PO_4, 85%), and sulfuric acid (H_2SO_4, 98%) were purchased from Beijing Chemical Works, China. Deionized water was used throughout the experiments.

1.2 preparation of nZVI

nZVI was synthesized with a classic method of chemical reduction, as previously reported [1]. Briefly, 100 mL 0.5 M of a $NaBH_4$ aqueous solution was added dropwise into 200 mL 0.045 M $FeCl_3 \cdot 6H_2O$ aqueous solution by peristaltic pump (revolving speed 20 rpm) at an ambient temperature with strong mechanical agitation. Fe(III) was reduced to Fe(0), according to the following reaction (Equation 1) [2]:

$$Fe(H_2O)_6{}^{3+} + 3BH_4^- + 3H_2O \rightarrow Fe^0 \downarrow + 3B(OH)_3 + 10.5H_2 \quad (1)$$

The process was proceeded by sparking with pure nitrogen (99.99%), which was stopped half an hour after adding the $NaBH_4$ aqueous solution. Afterwards, the mixture was settled for 1 h, and then the precipitate was washed several times by deoxy-deionized water and ethanol. Finally, the solid obtained was dried by vacuum freeze-drying (SiHuan, Beijing, China) and preserved in a seal.

2. Reactivity test

Table S1. PSO kinetic parameters of nZVI/rGO coupled with ultrasonic pre-treatment.

System	Equation	k_{ad} (g mg^{-1} min^{-1})	q_e (mg g^{-1})	R^2
Aerobic	$t/q_t = 0.02606 + 0.01303t$	0.0065	76.75	0.9976
US/aerobic	$t/q_t = 0.00538 + 0.01318t$	0.032	75.87	0.9969
Anaerobic	$t/q_t = 0.0212 + 0.01236t$	0.0072	80.90	0.9959
US/anaerobic	$t/q_t = 0.00415 + 0.01219t$	0.036	82.03	0.9996

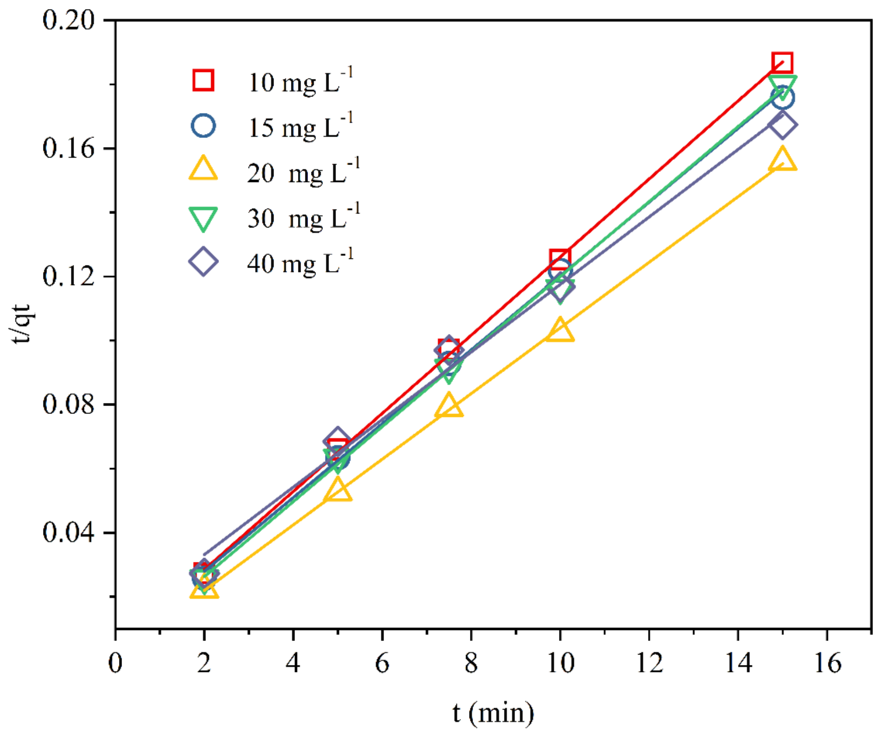

Figure S1. The fitted curves of the PSO model for US-nZVI/rGO system.

Table S2. Parameters obtained from PSO kinetic model for US-nZVI/rGO system.

Initial concentration (mg L^{-1})	Equation	k_{ad} (g mg^{-1} min^{-1})	q_e (mg g^{-1})	R^2
10	t/q_t = 0.00415 + 0.01219t	0.036	82.03	0.9996
15	t/q_t = 0.00495 + 0.01152t	0.027	86.81	0.9989
20	t/q_t = 0.00152 + 0.01024t	0.069	97.66	0.9997
30	t/q_t = 0.00292 + 0.01171t	0.047	85.40	0.9985
40	t/q_t = 0.01212 + 0.01054t	0.0092	94.88	0.9918

3. Characterization

3.1 EDS

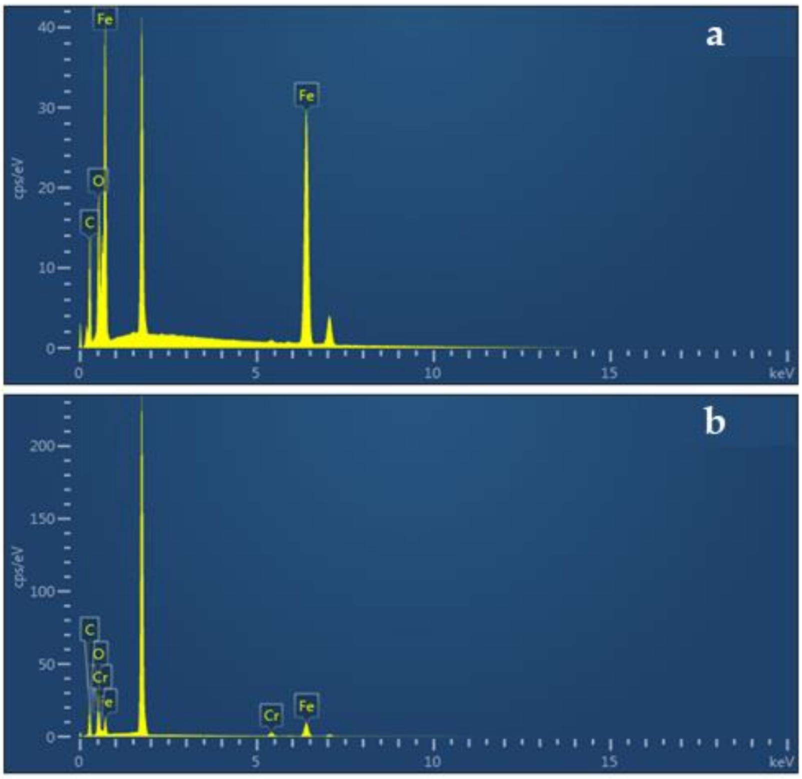

Figure S2. EDS spectra of a) nZVI/rGO and b) nZVI/rGO after reaction.

Table S3. The mass ratio of each element from EDS.

nZVI/rGO		nZVI/rGO after reaction	
elements	*wt%*	elements	*wt%*
C	15.18	**C**	26.22
O	7.20	**O**	28.97
Fe	77.62	**Fe**	39.17
		Cr	5.64
Total	100.00	Total	100.00

3.2 BET

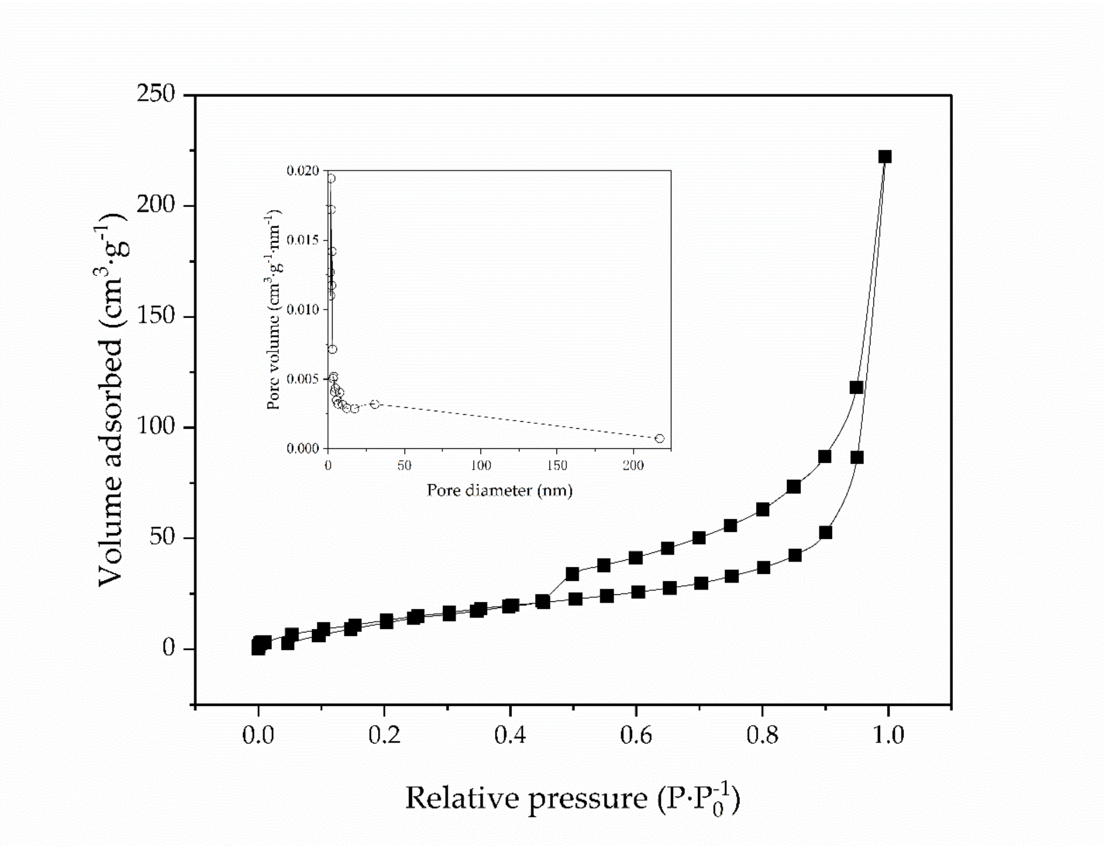

Figure S3. Nitrogen adsorption-desorption isotherms and pore size distribution curve (the insert pattern) for nZVI/rGO.

Table S4. Physical and chemical properties of nanoparticles investigated in this study.

**Sample**	**S_{BET}（m^2 g^{-1}）**	**Pore size（nm）**	**Pore volume（cm^3 g^{-1}）**	**Reference**
**GO**	1.23			this study
**nZVI**	14.56	4.12	0.11	previous study of our group[3]
**nZVI/rGO**	59.31	2.324	0.345	this study

Table S5. BET specific surface area of nZVI in the provious studies.

material	Synthesis method	Specific surface area ($m^2\ g^{-1}$)	Reference
**Nano Pd/Fe**	$NaBH_4$ reduction	33.5	[2]
**nZVI**	$NaBH_4$ reduction	24.4	[4]
**nZVI**	$NaBH_4$ reduction	25	[5]
**nZVI**	$NaBH_4$ reduction	14.2	[6]

3.3 XPS

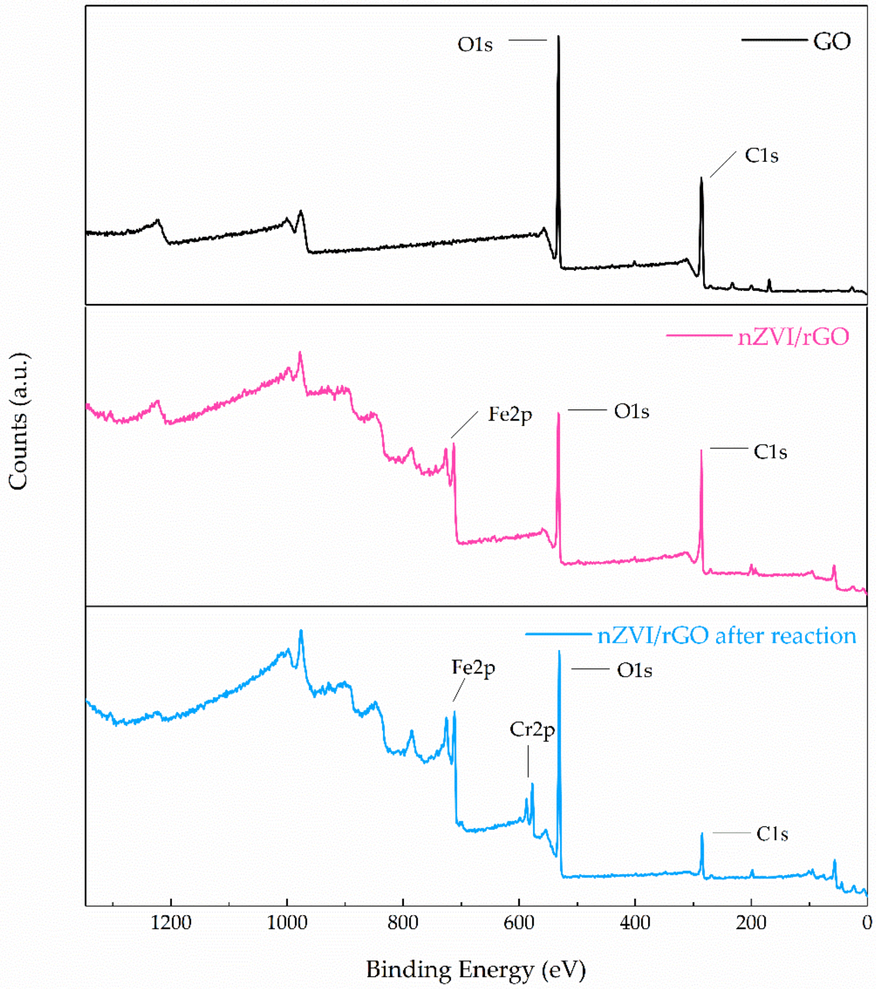

Figure S4. Full survey XPS patterns for nZVI/rGO, nZVI/rGO after reaction, and GO.

Table S6. The peak area fraction of carbon-containing groups.

**GO**		**nZVI/rGO**		**nZVI/rGO after reaction**	
**Groups**	Percentage (%)	Groups	Percentage (%)	Groups	Percentage (%)
**C-C**	44.06	C-C	62.45	C-C	63.13
**C-O**	6.62	C-O	20.76	C-O	20.96
**C=O**	40.32	C=O	9.15	C=O	4.92
**O-C=O**	9.00	O-C=O	7.65	O-C=O	10.99

4. Mechanism

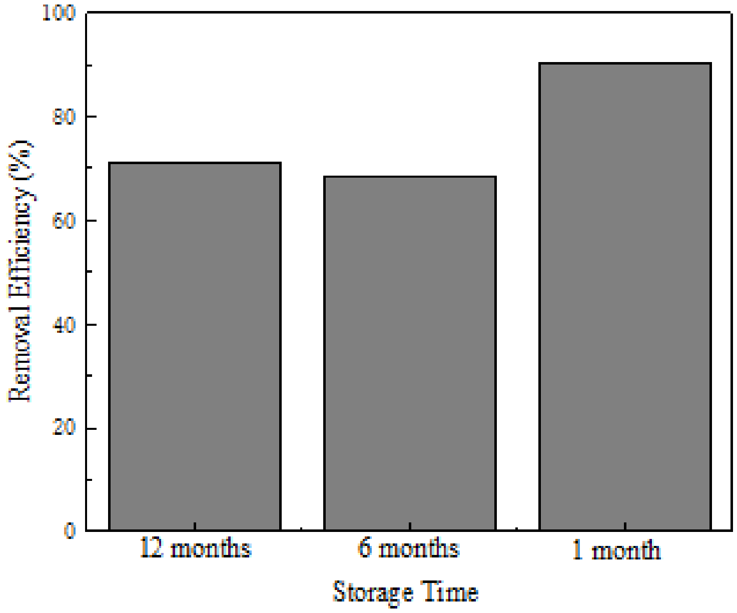

Figure S5. Influence of storage time.

Reference

1. Stefaniuk, M.; Oleszczuk, P.; Ok, Y.S. Review on nano zerovalent iron (nZVI): From synthesis to environmental applications. *Chemical Engineering Journal* **2016**, *287*, 618-632, doi:10.1016/j.cej.2015.11.046.
2. Wang C.B., Z.W.X. Synthesizing Nanoscale Iron Particles for Rapid and Complete Dechlorination of TCE and PCBs. *Environmental Science & Technology* **1997**, *31*, 2154-2156, doi:10.1021/es970039c.
3. Ma, Y.; Lv, X.; Yang, Q.I.; Wang, Y.; Chen, X. Reduction of carbon tetrachloride by nanoscale palladized zero-valent iron@ graphene composites: Kinetics, activation energy, effects of reaction conditions and degradation mechanism. *Applied Catalysis A: General* **2017**, *542*, 252-261, doi:10.1016/j.apcata.2017.05.028.
4. Kanel, S.R.; Manning, B.; Charlet, L.; Choi, H. Removal of arsenic(III) from groundwater by nanoscale zero-valent iron. *Environ Sci Technol* **2005**, *39*, 1291-1298, doi:10.1021/es048991u
5. Kanel, S.R.G., J. M.; Choi, H. Arsenic(V) Removal from Groundwater Using Nano Scale Zero-Valent Iron as a Colloidal Reactive Barrier Material. *Environ Sci Technol* **2006**, *40*, 2045-2050, doi:10.1021/es0520924
6. Uzum, C.; Shahwan, T.; Eroglu, A.E.; Lieberwirth, I.; Scott, T.B.; Hallam, K.R. Application of zero-valent iron nanoparticles for the removal of aqueous Co^{2+} ions under various experimental conditions. *Chem Eng J* **2008**, *144*, 213-220, doi:10.1016/j.cej.2008.01.024.
